# Supplementary material for: Patterns of Intron Gain and Loss in Fungi
Source: PLoS Biol. 2004 Nov 30;2(12):e422. doi: 10.1371/journal.pbio.0020422 (PMC532390; doi:10.1371/journal.pbio.0020422)
Supplement: Table S1 — Also available at http://genes.mit.edu/NielsenEtAl/. (4.3 MB ZIP). [file pbio.0020422.st001.zip › NielsenEtAl/html/257.html]

AN4388.1.NCU08940.1.MG06192.1.FG10293.1


```
 CLUSTAL W (1.82) Multiple Sequence Alignments - Introns Inserted


Sequence 1: NCU08940.1	123 aa
Sequence 2: FG10293.1	123 aa
Sequence 3: AN4388.1	128 aa
Sequence 4: MG06192.1	126 aa
Alignment Length: 130 aa
Number Identitical Residues: 45 aa
Alignment Score (without introns) 2630


MG06192.1 	MSYPSAAPFILRRPWLKKLLMPVAEWHANAAGYRQMGLR2ADDLIPEESETVQAAIKRLD
NCU08940.1	MSAPSLVQAVAKRGWLNKMLKPVSNWYINAAGYRQMGLR2ADDLISEENETVLAALQRLS
FG10293.1 	-MKYSLAPFILRRPWLARFFKPAATWYVNTAGYRQMGLK2YDDLLEEENETAQAALKRLS
AN4388.1  	MSAPSLTSYIVKRPWLKRWMMPIANWYTDAAGYRKLGLK2ADDLIPEESDTVQTALKRLP
          	    * .  : :* ** : : * : *: ::****::**:  ***: **.:*. :*::** 

MG06192.1 	AKENYDRIFRLRRAAQL---~-SLSHQLLPKSEWTKPEQ~DVPYLLPIIKRVEAELAEKA
NCU08940.1	PKESYDRIYRIRRATQL---~-SLTHKLLPKNEWTTQEE~DVPYLRPLIDQIEAEAAEKD
FG10293.1 	NRESYERIYRIRRAVQC---~-SYQHKLLPKDQWTTSAA0DKPYLQPLMEEVAAEKAEKN
AN4388.1  	PKEAYDRIFRIRRAFQVGFI0CSVSHTLLPPEEHIKPNE0DVEYLSPIIREIEKENAERA
          	 :* *:**:*:*** * .   .*  * *** .:  .    *  ** *:: .:  * **: 

MG06192.1 	ALDSAEVLPKGAH
NCU08940.1	ALDTLAVVKN---
FG10293.1 	ELDSIAVVRKH--
AN4388.1  	DLDNLVVNRKK--
          	 **.  *  :
```
